# Protection and Response of a Tertiary Hospital in South Korea to the COVID-19 Outbreak

**DOI:** 10.1017/dmp.2020.199

**Published:** 2020-06-22

**Authors:** Hye Jin Shi, Jae Back Lee, Min Kyung Choi, Young-Rock Jang, Yong-Kyun Cho, Joong Sik Eom

**Affiliations:** Division of Infectious Diseases, Department of Internal Medicine, Gil Medical Center, Gachon University School of Medicine, Incheon, Republic of Korea; Unit of Infection Control, Gil Medical Center, Gachon University School of Medicine, Incheon, Republic of Korea

**Keywords:** COVID-19, protection, response, South Korea, tertiary hospital

## Abstract

**Objective:**

Here, we present an overview of how a tertiary hospital responded to maintain necessary activities and protect patients and staff from the coronavirus disease (COVID-19) outbreak.

**Methods:**

Gil Medical Center, a tertiary hospital in Incheon, has operated a special response team since January 21, 2020. All visitors were assessed for body temperature and respiratory symptoms, and screened for recent overseas travel. Suspected COVID-19 patients were taken to a screening clinic. All febrile patients with or without respiratory symptoms were taken to a respiratory safety clinic. An isolation ward, which consisted of 10 negative-pressure rooms, was used to treat confirmed cases. More than 120 beds were prepared for the outbreak, and patients with pneumonia were preemptively isolated.

**Results:**

By May 5, 480 960 visitors were assessed at the control station, 3350 patients visited the triage center, and 1794 were treated in the respiratory safety clinic. Seventeen confirmed cases were admitted to the negative isolation ward, and 350 patients with pneumonia were preemptively isolated. A total of 2977 severe acute respiratory syndrome coronavirus 2 (SARS-CoV-2) polymerase chain reaction tests were performed.

**Conclusions:**

While tertiary hospitals play an important role in treating both COVID-19 patients and non-COVID-19 patients, hospital staff have to protect themselves from unexpected in-hospital transmission. A multifaceted response must be undertaken to protect tertiary hospitals and their staff during the COVID-19 epidemic.

Severe acute respiratory syndrome coronavirus 2 (SARS-CoV-2), the virus that causes coronavirus disease (COVID-19), had a global impact, and the World Health Organization (WHO) declared the disease a pandemic.^1^ By May 5, 2020, there were 3 517 345 laboratory confirmed-cases, but Korea had only 10 806 confirmed cases, despite large test numbers.^2,3^ The world had a lot of questions about how Korea controlled its COVID-19 outbreak.^4^

During the epidemic, tertiary hospitals in Korea screened cases, isolated patients, and treated confirmed severe cases. Gil Medical Center (GMC) is a 1450-bed, tertiary care hospital in Incheon, Republic of Korea, and operates with unique surveillance and isolation systems for COVID-19. As a result of these efforts, the hospital experienced no outbreaks and continued to function effectively over the 3-month course of the COVID-19 epidemic. We describe our response and protective strategies for controlling the epidemic in a Korean tertiary care hospital with regard to the timeline of the outbreak, with the aim of providing recommendations for controlling future COVID-19 epidemics.

## METHODS

We used all records created during the outbreak, including patients’ contact investigation records, monitoring records for all admitted suspected or confirmed cases, results of daily SARS-CoV-2 polymerase chain reaction (PCR) assays, and the number of triage center outpatients, quarantined persons, and COVID-19 cases from January 21 to May 5, 2020. Various control measures were implemented in response to the critical events specific to each phase of the epidemic. This study was approved by the institutional review board of the GMC.

### Changes in COVID-19 Case Definition

The Korea Center for Disease Control (KCDC) provides definitions of COVID-19 confirmed cases, suspected cases, and patients under investigation (PUI). From January 21 to March 2, the KCDC made 4 major changes to the definition of suspected cases and PUI to apply epidemiologic change.

### Isolation Ward

The GMC had 1 isolation ward, with 10 negative-pressured rooms. It was a nationally designated isolation ward and controlled by government and GMC infection specialist co-operations. After receiving a declaration of suspected cases or confirmed cases (ie, from airport quarantine or a general hospital), the local government made transfer requests to GMC. The suspected case was transferred to GMC through a negative-pressure ambulance and an independent elevator directly to the isolation ward from outside. GMC infectious disease specialists wearing personal protective equipment (PPE) examined admitted patients and performed tests for SARS-CoV-2 and chest radiography (ie, CXR).

### Triage Center and Respiratory Safety Clinic

GMC began operating as an independent triage center on January 26, 2020. Outpatients and visitors to the emergency department (ED) with epidemiologic links or with respiratory symptoms were treated at the triage center. The first triage center was located at the ED and consisted of 1 clinic, 1 radiography room, 2 sample collection rooms, and 1 waiting room, all of which were negatively pressurized and separated from other ED spaces. After a large outbreak occurred at February 24, we expanded the respiratory safety clinic at a separate building and established 6 negative-pressure offices.

### Entrance Control Station

GMC had 43 entrances, and it was impossible to monitor all of the entrances. We blocked all entrances except for the 10 entrances and installed control stations. Security staff, nurses, and health care workers (HCWs) in other service departments were equipped with PPE and stationed at the entrances to check all hospital visitors, including patients, HCWs, caregivers, visitors, and suppliers, for symptoms and epidemiologic risks.

### HCW Monitoring and Work Restrictions

Community transmission began in Korea on February 18, and we performed contact tracing for our HCWs. We assessed for visits to danger zones within the previous 2 weeks, possible contact with confirmed cases, and the presence of symptoms.^5,6^ If an HCW developed symptoms with an epidemiologic link, that individual was immediately placed under work restrictions until a negative result for SARS-CoV-2 was obtained.

## RESULTS

### Changes in COVID-19 Case Definition

As defined by the KCDC, confirmed cases were those with the presence of infectious pathogens regardless of clinical presentation; this definition did not change during the study period. Suspected cases were first restricted to visitors of Wuhan city who had symptoms, but the epidemiology changed and the spectrum of suspected cases also expanded to include patients who developed a fever (> 37.5°C) or respiratory symptoms (eg, cough, dyspnea, sore throat) within 14 days of contact with a confirmed patient during the period of symptomatic manifestation. The definition of PUI changed most dramatically, from pneumonia with a history of visiting China to solely pneumonia (Table 1).

**TABLE 1:** Changes to COVID-19 Case Definitions in Korea

Category	3^rd^ Definition	4^th^ Definition	5^th^ Definition	7^th^ Definition
Date	January 21	January 27	February 7	March 2
CC	Confirmation of infectious pathogen (SARS-CoV-2) regardless of clinical pattern			
SC	Fever* or respiratory symptom(s)** + close contact with confirmed cases in the previous 14 days			
	Pneumonia or suspected pneumonia + a visit to Wuhan city in the previous 14 days	Fever or respiratory symptom(s) + a visit to Hubei Province in the previous 14 days	Fever or respiratory symptom(s) + a visit to China in the previous 14 days	
			Doctor’s opinion of suspected COVID-19 (eg, unexplained pneumonia, fever, or respiratory symptoms after visiting a local transmission area***)	
PUI	Fever and respiratory symptom(s) + a visit to Wuhan city in the previous 14 days	Pneumonia + a visit to China in the previous 14 days		Doctor’s opinion of suspected COVID-19 (eg, unexplained pneumonia)
				Fever or respiratory symptom(s) + a visit to a country with possible local transmission
				Fever or respiratory symptom(s) + an epidemiologic link with the domestic outbreak in the previous 14 days

*Notes:* *fever = body temperature over 37.5°C; **respiratory symptom(s) = cough, dyspnea, sputum, sore throat, etc.; *** local transmission area = updated daily by the World Health Organization (WHO); CC = confirmed cases; SC = suspected cases; PUI = patient under investigation; SARS-CoV-2 = severe acute respiratory syndrome coronavirus 2; COVID-19 = coronavirus disease 2019.

Regions of local transmission (danger zones) were the key to case definitions in GMC. Before February 7, the danger zone was limited to the city of Wuhan and Hubei Province, China. After February 7, when an epidemic outside of China was reported, GMC updated our danger zone daily according to the WHO situation report. After February 18, when the Daegu city, Gyeongsangbuk Province, and Shincheonji (religious group) outbreaks occurred in South Korea, GMC rapidly expanded the danger zone to Daegu city and involvement with Shincheonji. After March 8, when Seoul and the metropolitan area were in danger, we put less emphasis on geography and more on symptoms and contact with confirmed cases.

### Isolation Wards

On February 24, the first confirmed case (a 70-year-old man diagnosed for COVID-19 at a nursing hospital in Gyeongsangbuk Province) was transferred to GMC due to the lack of an isolation room in the local area. We have 1 isolation ward, but it was vital to separate confirmed cases from suspected cases or PUI. Hospital management decided to empty 1 floor for suspected cases. A 46-bed ward with portable negative-pressure devices was opened on February 28, and suspected cases or PUI from the triage center or outpatient clinic were admitted to this ward.

By May 5, 17 confirmed cases with 5 recovered cases (Table 2) had been admitted to the isolation ward. A total of 350 suspected cases or PUI were admitted to the second isolation ward, with no confirmed cases among them.

**TABLE 2:** Epidemiologic Information About Confirmed Cases Admitted to GMC

N	Age/Sex	Nationality	Confirmed Date	Location	Source of Infection	Initial Severity	Admission Date	Discharge Date	Underlying Disease(s)	Clinical Outcome	Latest RT-PCR
1	70/M	Korea	21 February	Daegu	Nursing hospital outbreak	Moderate	24 February	16 March	Schizophrenia	Symptoms relieved	Negative (2 times)
2	56/M	Korea	24 February	Daegu	Shincheonji	Mild	28 February	28 March	CKD on HD	Symptoms relieved	Negative (2 times)
3	76/M	Korea	28 February	Gimcheon	Close contact with confirmed case	Very severe	01 March	01 April	Prostate cancer, diabetes mellitus	Symptoms relieved	Negative (2 times)
4	70/F	Korea	20 February	Sangju	Nursing hospital outbreak	Severe	04 March	TBD	Dementia	On ventilator, CRRT	Negative (2 times)
5	50/M	Korea	06 March	Incheon	Nursing hospital outbreak	Asymptomatic	06 March	TBD	None	Asymptomatic	Negative (1 times)
6	93/F	Korea	08 March	Andong	Shincheonji	Severe	09 March	31 March	Dementia	Symptoms relieved	Negative (2 times)
7	38/F	Korea	14 March	Incheon	Telephone marketing outbreak	Mild	14 March	TBD	None	Symptoms relived	Positive
8	49/M	Philippines	20 March	Airport	Imported	Mild	20 March	TBD	None	On ventilator, ECMO, CRRT	Negative (1 times)
9	56/F	Korea	28 March	Airport	Imported	Asymptomatic	28 March	TBD	None	Asymptomatic	Positive
10	47/F	Korea	28 March	Incheon	Close contact with confirmed case	Asymptomatic	28 March	TBD	None	Asymptomatic	Positive
11	76/M	U.S.	30 March	Incheon	Imported	Mild	30 March	TBD	Prostate cancer	Symptoms relieved	Positive
12	47/F	Philippines	1 April	Incheon	Close contact with confirmed case	Asymptomatic	1 April	TBD	None	Asymptomatic	Positive
13	7/F	Philippines	1 April	Incheon	Close contact with confirmed case	Asymptomatic	1 April	TBD	None	Asymptomatic	Positive
14	67/F	Korea	1 April	Incheon	Close contact with confirmed case	Mild	1 April	25 April	Hypertension	Symptoms relieved	2 Negative
15	70/M	Korea	4 April	Incheon	Imported	Asymptomatic	4 April	TBD	COPD	Asymptomatic	Positive
16	67/F	Korea	9 April	Seoul	Imported	Asymptomatic	9 April	TBD	Hypertension	Asymptomatic	1 Negative
17	82/M	Korea	29 April	Incheon	Imported	Very severe	29 April	TBD	Atrial fibrillation, hypertension	On high dose O2 inhalation	Positive

*Notes:* GMC = Gil Medical Center; N = number; US = United States; TBD = to be determined; CKD = chronic kidney disease; HD = hemodialysis; CRRT = continuous renal replacement therapy; ECMO = extracorporeal membrane oxygenation; RT-PCR = reverse transcription polymerase chain reaction; COPD = chronic obstructive pulmonary disease.

### Triage Center

On January 26, the triage center was built within the ED. A nurse wearing PPE took a simple history of the patient and reception was made by telephone. During each visit, the patient’s epidemiologic risk factors and symptoms were recorded. A doctor with PPE examined the patient, and a CXR was performed in all patients. If a suspected case or PUI required admission, the patient was placed in the isolation ward. By May 5, 3350 patients visited the triage center, and 1 patient was confirmed to have COVID-19. However, no further exposure occurred because of complete separation of the areas and hygiene protocols.

### Respiratory Safety Clinic

As local transmission in Korea became a reality and large hospitals were shut down, we tried to be especially careful about separating suspected cases and PUI from other patients. We opened the respiratory safety clinic at a different building on February 24. Patients with any symptoms had mandatory assessments at the respiratory safety clinic or triage center before they reached the hospital. The triage protocol here was identical to that of the triage center. By May 5, 1794 patients had been treated in the safety clinic.

### Entrance Control Station

HCWs at 10 control stations screened visitors for symptoms (eg, fever, respiratory symptoms), recent visits to danger zones, history of contact with confirmed cases, and the purpose of their visit. The national drug utilization review temporarily provides information on visits to danger zones (countries in which local transmission occurred), making it possible to double check. All unnecessary visitations were rejected.

People with symptoms or a history of visits to risky areas were referred to the triage center via an outside pathway by security staff wearing PPE. By May 5, 480 960 people were assessed at the control station, and no unprotected exposures to confirmed cases or suspected cases occurred at GMC.

### Number of SARS-CoV-2 Tests

From January 21 to February 7, only 27 tests were performed at the GMC. Before February 7, real-time PCR kits for testing for SARS-CoV-2 were only available through the KCDC, and doctors could not perform this test without government permission. After February 7, the SARS-CoV-2 testing was expanded to private medical institutions. A total of 2977 tests were performed at the GMC by May 5.

### Personal Protective Equipment

Owing to the unknown pathophysiology of the disease, at first, all HCWs at the triage center or isolation wards wore head-covering coveralls (level D) and a powered air-purifying respirator (PAPR). HCWs working at the control center wore 5-piece protection (ie, gown, gloves, N-95 mask, goggles or face shield, hair cap).

After February 18, the Daegu area experienced a critical shortage of equipment, and country-level downsizing of PPE was required. After March 8, we downgraded our hospital PPE level. Only HCWs dealing with confirmed cases wore level D protection and a PAPR. HCWs working at the triage center or at the control center wore 5-piece protection.

### HCW Monitoring and Work Restrictions

After March 8, when transmission in the metropolitan area occurred, 14 HCWs were monitored. They all tested negative, and 1 HCW who had visited the Daegu area was placed under work restrictions because of persistent symptoms.

## DISCUSSION

Tertiary hospitals in Korea had to play a vital role in managing the COVID-19 epidemic. They take the lead in both accepting confirmed cases and handling primarily suspected cases during outbreaks. At the same time, they had to play a primary role with treating severe, critically ill patients (including the immune-suppressed), as well as quarantine gate their local regions, screening all pneumonia patients. The exposure to unscreened patients in hospitals posed many problems, specifically the additional propagation of the disease by causing mass outbreaks in high-risk patients^7,8^ and exposing HCWs to risk. When in-hospital exposure occurs, hospitals cannot function effectively, and it is impossible to conduct necessary medical treatments. In Korea, some hospitals had to close temporarily because of in-hospital transmission of COVID-19.^9^

Hospital protection may need multiple strategies simultaneously. In almost every case, especially during an epidemic or pandemic, dealing with a newly emerging infectious disease is very challenging because of lack of experience, knowledge, and preparation. We wanted to share our experiences because our situation is similar or perhaps more serious than in other countries suffering from COVID-19.

Based on our experience, changing both the targets and strategies based on the degree of local transmission is a key control measure in the COVID-19 epidemic. When local transmission in Incheon was uncommon, additional non-targeted screening measures, including symptom monitoring and CXR screening, did not detect additional patients with COVID-19 without epidemiologic links. In addition, quarantine played an important role, and defining an accurate epidemiologic danger zone and assessing for epidemiologic links were key components to finding additional patients. We expanded or narrowed the definition of suspected cases and PUI using up-to-date information from the KCDC.

Total isolation of the pathways taken by PUI was also a key component. COVID-19 patients are often indistinguishable from non-patients based on symptoms or CXRs because of the highly contagious nature and nonspecific symptoms of COVID-19.^7,10^ During the epidemic, we operated a ward only for suspected cases or PUI, so that we could almost completely separate these cases from other inpatients. Though there was only 1 confirmed case out of 3300 triage visits, the vital role of the triage center was to reduce exposures to COVID-19 within the hospital. Total separation of pathways taken by PUI and non-patients was also helpful during contact investigations. Two potential exposures occurred during the study period. However, there were no further exposures in either 2 cases, and we did not need to perform contact tracing inside our hospital. Pathway separation was especially important during active local transmission, when the number of patients increased and it was necessary to guard against massive local transmission.

## CONCLUSION

In conclusion, hospitals and their staff have to concentrate on protecting themselves from unexpected in-hospital transmission during the COVID-19 epidemic. The changing of target individuals with epidemiologic links according to the degree of local transmission, operating the triage center, controlling hospital entry, preparing capacity to isolation, and adjusting the level of quarantine and PPE according to the expected degree of local transmission were all strongly important to protect hospitals and countries during the COVID-19 pandemic.
